# Clonal Hematopoiesis and Cardiovascular Disease Risk After Cancer Therapy in Patients With Solid Tumors

**DOI:** 10.1001/jamaoncol.2025.5785

**Published:** 2026-01-08

**Authors:** Derek Shyr, Yash Pershad, Kun Zhao, Ashwin Kishtagari, Robert W. Corty, Eric Shinohara, Ben Ho Park, J. Brett Heimlich, Leo Luo, Alexander G. Bick

**Affiliations:** 1 Heersink School of Medicine, The University of Alabama at Birmingham, Birmingham; 2 Division of Genetic Medicine, Department of Medicine, Vanderbilt University Medical Center, Nashville, Tennessee; 3 Division of Hematology Oncology, Department of Medicine, Vanderbilt University Medical Center, Nashville, Tennessee; 4 Division of Rheumatology, Department of Medicine, Vanderbilt University Medical Center, Nashville, Tennessee; 5 Department of Radiation Oncology, Vanderbilt University Medical Center, Nashville, Tennessee; 6 Division of Cardiovascular Medicine, Department of Medicine, Vanderbilt University Medical Center, Nashville, Tennessee

## Abstract

**Importance:**

Cancer survivors have increased cardiovascular disease (CVD) risk partly due to the toxic effects of cancer therapy. Clonal hematopoiesis of indeterminate potential (CHIP), an age-associated blood disorder caused by somatic variants in blood stem cells, is more prevalent among individuals receiving cancer therapy and increases CVD risk independent of traditional risk factors. It is unknown whether CHIP amplifies therapy-related cardiovascular toxic effects in patients with cancer.

**Objective:**

To assess the association between CHIP and CVD risk, accounting for competing risks, among patients with primary solid tumors who received chemotherapy, radiotherapy, or immunotherapy.

**Design, Setting, and Participants:**

A cohort study was conducted using BioVU, Vanderbilt University Medical Center’s biorepository linking electronic health records to whole-genome sequencing data from 250 038 participants from 2006 to 2025. In this cohort, participants had a primary solid tumor diagnosis, received chemotherapy, radiotherapy, and/or immunotherapy, and did not have hematologic malignant disease before treatment. Data were analyzed from June 2025 to November 2025.

**Exposures:**

CHIP variants detected via whole-genome sequencing, chemotherapy, radiotherapy, and immunotherapy.

**Main Outcomes and Measures:**

Time to first cardiovascular event, defined as heart failure, ischemic CVD, or arrhythmia following cancer treatment.

**Results:**

Among 8004 eligible participants (median [IQR] age, 61.9 [52.2-69.9] years; 4385 female individuals [54.8%]) with a primary solid tumor diagnosis, 7438 had no heart failure, 7392 no ischemic CVD, and 6002 no arrhythmia before cancer therapy. Overall, 549 (6.9%) had CHIP. In the propensity score–matched cohort, participants with CHIP had a significantly higher 10-year cumulative incidence of heart failure (20.3%; 95% CI, 16.0%-24.4% vs 14.5%; 95% CI, 13.5%-15.6%; *P* = .001) and ischemic CVD (25.3%; 95% CI, 20.5%-30.0% vs 18.5%; 95% CI, 17.3%-20.0%; *P* < .001) compared with those without CHIP. In adjusted Fine-Gray models, CHIP was associated with increased risk of heart failure (subdistribution hazard ratio [sHR], 1.26; 95% CI, 1.02-1.56; *P* = .03). In an exploratory 24-month landmark analysis, there was a statistically significant interaction between CHIP and intensive chemotherapy (≥7 cycles) on heart failure risk (sHR, 1.02; 95% CI, 1.00-1.04; *P* = .03).

**Conclusions and Relevance:**

In this cohort study, CHIP was associated with increased CVD risk in patients with solid tumors receiving cancer therapy. This finding suggests incorporating CHIP status may improve cardio-oncology treatment of cancer survivors.

## Key Points

**Question:**

Is clonal hematopoiesis of indeterminate potential (CHIP) associated with increased risk of therapy-related cardiovascular disease among patients receiving chemotherapy, radiotherapy, or immunotherapy?

**Findings:**

In this cohort study of 8004 participants, CHIP was associated with higher risk of heart failure in patients receiving cardiotoxic cancer therapies, with effects more pronounced among those receiving higher cumulative doses of chemotherapy.

**Meaning:**

CHIP testing may improve cardiovascular risk stratification in oncology patients and support earlier cardio-oncology consultation, monitoring, and risk-based primary prevention strategies for high-risk cancer survivors.

## Teaser

This cohort study examines the association between clonal hematopoiesis of indeterminate potential and cardiovascular disease risk among patients with primary solid tumors who received chemotherapy, radiotherapy, or immunotherapy.

## Introduction

Cardiovascular disease (CVD) is the leading cause of noncancer deaths among cancer survivors, with chemotherapy and immunotherapy contributing to this excess risk.^1,2,3,4^ Clonal hematopoiesis of indeterminate potential (CHIP), an age-associated phenomenon that occurs when somatic variants in preleukemic genes are harbored in 4% or more of peripheral nucleated blood cells, independently increases CVD risk.^5,6^ Because patients with solid tumors demonstrate higher CHIP prevalence than age-matched controls,^7,8^ understanding whether CHIP amplifies therapy-related cardiovascular toxic effects has immediate clinical implications for risk stratification and monitoring strategies in cancer survivors. We hypothesized that CHIP would be associated with increased CVD risk in patients receiving cardiotoxic cancer therapies. Because cumulative exposure to cancer therapies is a major determinant of cardiotoxic effects, we further hypothesized that CHIP amplifies this risk, with a greater effect among those receiving higher cumulative chemotherapy exposure.^9^

## Methods

### Study Design and Participants

**Figure 1.**
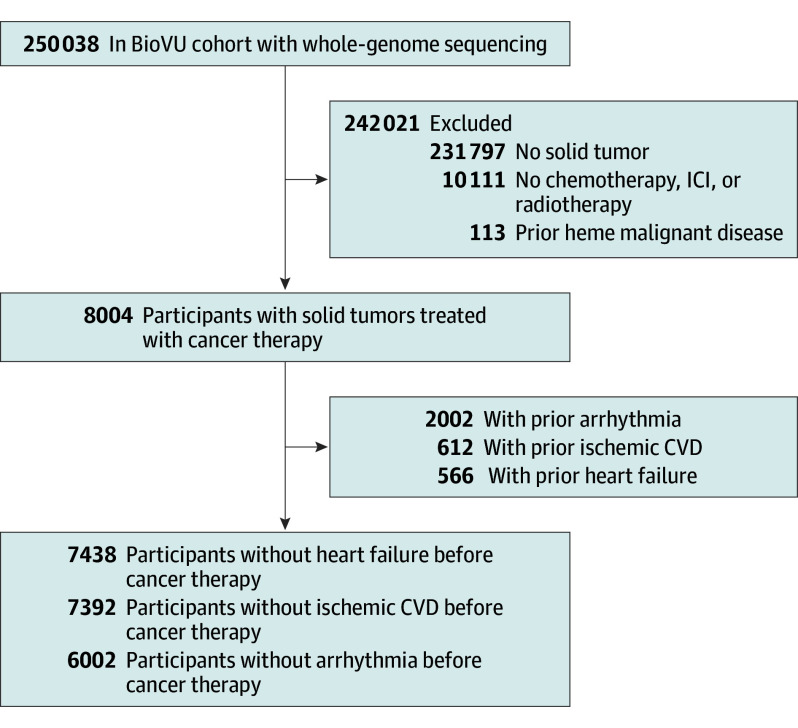
Flow Diagram of Cohort Inclusion Criteria Cancer therapy includes either chemotherapy, immune checkpoint inhibitors (ICIs), or radiotherapy. CVD indicates cardiovascular disease.

We conducted a cohort study using BioVU, the Vanderbilt University Medical Center (VUMC) biorepository linking electronic health records to whole-genome sequencing data from 250 038 participants from 2006 to 2025.^10^ In our study, 8004 participants had a primary solid tumor diagnosis, received chemotherapy, radiotherapy, and/or immunotherapy (eTable 1 in Supplement 1), and did not have hematologic malignant disease before cancer treatment. Among these participants, 7438 had no heart failure, 7392 no ischemic CVD, and 6002 no arrhythmia before cancer therapy (Figure 1). The study was approved by VUMC’s institutional review board and followed the Strengthening the Reporting of Observational Studies in Epidemiology (STROBE) reporting guidelines.

### Clonal Hematopoiesis of Indeterminate Potential Detection

CHIP was defined as somatic variants in 58 established myeloid hematologic malignancy driver genes with a variant allele frequency of 2% or more in participants without hematologic malignant disease. Variants were identified using whole-genome sequencing with variant calling performed using GATK Mutect2, version 4.3.0.0 (GATK), and ANNOVAR, version 2020-06-08 (Qiagen), annotation as previously described.^11^

### Outcomes

The primary outcome was time to first cardiovascular event, defined as heart failure, ischemic CVD, or arrhythmia following cancer treatment initiation. The definitions of these outcomes, including the specific diagnoses based on standard *International Classification of Diseases, Ninth Revision (ICD-9)* and *International Statistical Classification of Diseases and Related Health Problems, Tenth Revision (ICD-10)* codes, are provided in eTable 1 in Supplement 1.

### Statistical Analysis

To adjust for baseline covariate imbalance between participants with and without CHIP, we performed 1:10 propensity score matching using a logistic regression model including age, sex, self-reported race and ethnicity, tumor type, cancer treatment regimen, hypertension, and low-density lipoprotein (LDL) cholesterol as covariates. Covariate balance after matching was assessed using absolute standardized mean differences. Missing data were handled using multiple imputation by chained equations.^12^ Cumulative incidence curves for CVD stratified by CHIP status were estimated using the Fine-Gray competing risks method.^13^ We used 2-sided Gray tests at a significance level of *P* < .05 to compare cumulative incidence curves. Multivariable Fine-Gray models were used to assess the association between CHIP and the subdistribution hazard of CVD, adjusting for age, sex, self-reported race and ethnicity, cancer treatment regimen, hypertension, and LDL cholesterol. Exploratory landmark analyses examining the interaction between CHIP and cumulative exposure to chemotherapy based on the number of cycles were conducted using Fine-Gray competing risks models at 12, 18, and 24 months. Clinically, these time points reflect early, intermediate, and extended periods of chemotherapy exposure in oncology practice.^14,15^ Statistically, we selected these time points to ensure a sufficient number of events within each window for stable estimation of the subdistribution hazard. Because the number of chemotherapy cycles varies by cancer type and regimen, we predefined intensive chemotherapy as 7 or more cycles, a threshold above the typical 4 to 6 cycles used in standard solid tumor regimens.^16,17,18^ We used 2-sided Gray tests at a significance level of *P* < .05 to compare outcomes between participants with and without CHIP. All analyses were performed using R (version 4.5.0., R Foundation). Data were analyzed from June 2025 to November 2025.

## Results

### Study Population

Descriptive characteristics of eligible participants from BioVU were summarized in eTable 2 in Supplement 1. Among 8004 participants, 549 (6.9%) had CHIP. Participants with CHIP were older (median [IQR] age, 69.8 [62.3-75.7] vs 61.3 [51.6-69.3] years), more likely to be male (298 of 549 [54%] vs 3321 of 7455 [45%]), and have hypertension (418 of 538 [78%] vs 5086 of 7330 [69%]) compared with those without CHIP. After 1:10 propensity score matching, baseline characteristics (Table) were well balanced with absolute standardized mean differences lower than 0.1 (eFigure 1 in Supplement 1).

**Table.** Demographic and Clinical Profiles After Multiple Imputation and Propensity Score Matching

Characteristic	No. (%)	
	No CHIP (n = 7367)	CHIP (n = 542)
Age, median (IQR), y	61.3 (51.6-69.3)	69.7 (62.3-75.7)
Sex		
Female	4089 (56)	248 (46)
Male	3278 (44)	294 (54)
Race		
Asian	82 (1.1)	4 (0.7)
Black	842 (12)	53 (9.8)
Indian/Alaska Native	9 (0.1)	0
White	6358 (87)	484 (89)
Ethnicity		
Hispanic	166 (3.0)	11 (2.6)
Not Hispanic	5412 (97)	417 (97)
Ever smoked		
No	4637 (63)	321 (59)
Yes	2730 (37)	221 (41)
Hypertension		
No	2245 (30)	121 (22)
Yes	5122 (70)	421 (78)
LDL levels, median (IQR), mg/dL	102.7 (95.0-105.0)	102.7 (88.0-102.7)
Primary solid tumor		
Breast	2332 (32)	118 (22)
Gastrointestinal	2065 (28)	153 (28)
Genitourinary	2231 (30)	213 (39)
Lung	140 (1.9)	15 (2.8)
Melanoma	595 (8.1)	42 (7.7)
Thyroid	4 (<0.1)	1 (0.2)
Chemotherapy type		
Alkylating agent/mitomycin	208 (3.8)	14 (3.5)
Anthracycline	357 (6.6)	22 (5.6)
Nitrogen mustard	395 (7.3)	27 (6.8)
Other	1181 (22)	89 (23)
Platinum compound	1071 (20)	70 (18)
Pyrimidine analog	952 (18)	91 (23)
Taxane	1052 (19)	69 (17)
TKI	224 (4.1)	13 (3.3)
Chemotherapy cycles, median (IQR)	4 (2-9)	3 (2-8)
Chemotherapy		
No	1927 (26)	147 (27)
Yes	5440 (74)	395 (73)
Radiotherapy		
No	3727 (51)	285 (53)
Yes	3640 (49)	257 (47)
Immune checkpoint inhibitors		
No	6860 (93)	510 (94)
Yes	507.0 (6.9)	32.0 (5.9)

Abbreviations: CHIP, clonal hematopoiesis of indeterminate potential; LDL, low-density lipoprotein cholesterol; TKI, tyrosine kinase inhibitor. SI conversion factor: To convert LDL to mmol/L, multiply by 0.0259.

### Cardiovascular Outcomes

In the propensity score-matched cohort, participants with CHIP had significantly higher 10-year cumulative incidence of heart failure compared with those without CHIP (20.3%; 95% CI, 16.0%-24.4% vs 14.5%; 95% CI, 13.5%-15.6%; *P* = .001). A similar pattern was observed for ischemic CVD (25.3%; 95% CI, 20.5%-30.0% vs 18.5%; 95% CI, 17.3%-20.0%; *P* < .001).

**Figure 2.**
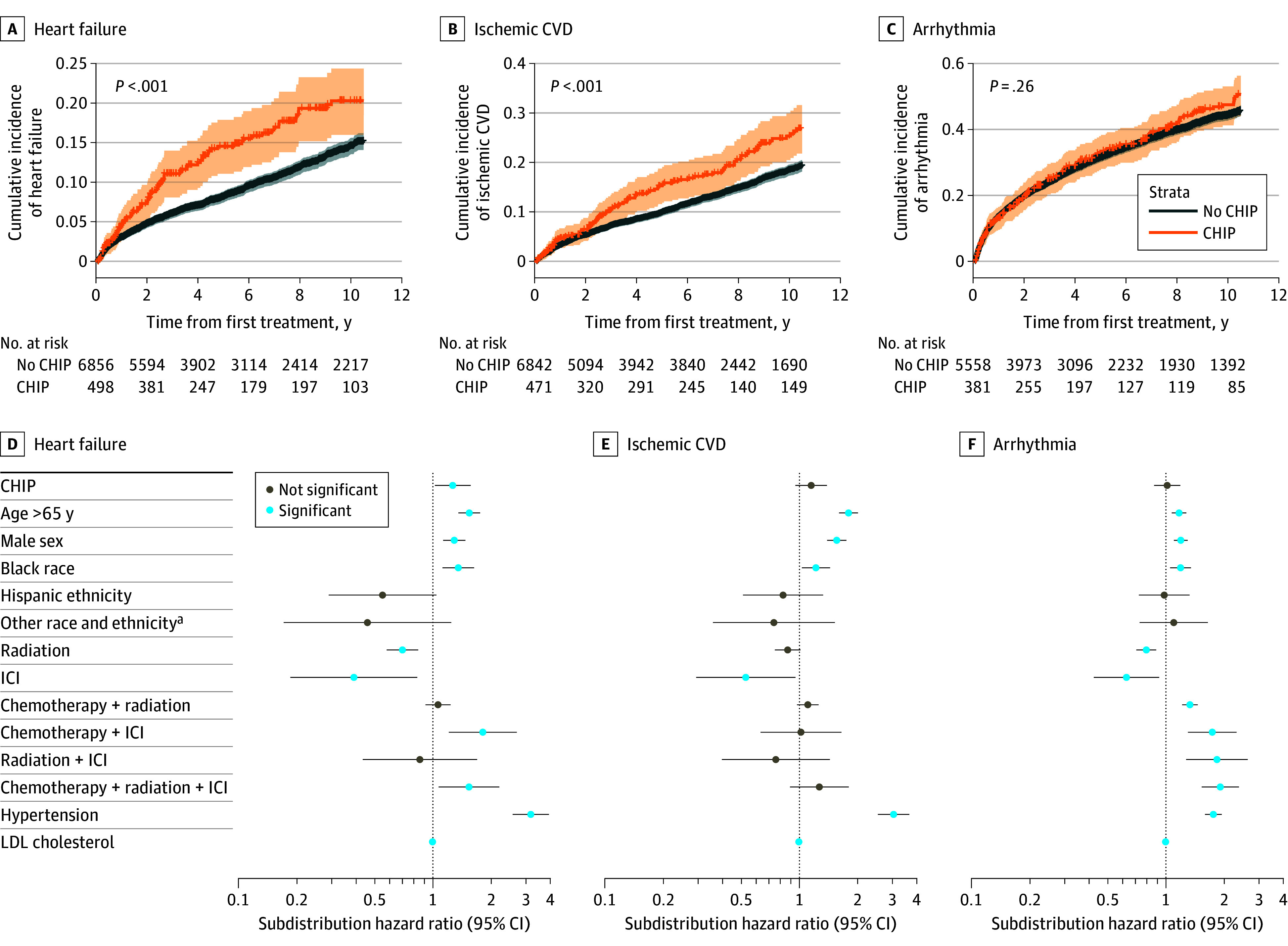
Cumulative Incidence and Adjusted Risk of Cardiovascular Disease (CVD) by Clonal Hematopoiesis of Indeterminate Potential (CHIP) Status After Propensity Score Matching A, B, and C, Cumulative incidence plots comparing CHIP (orange) to no CHIP (blue). D, E, and F, Dot plots of subdistribution hazard ratios from multivariable analysis. Reference levels of categorical covariates included age (≤65 years), sex (female), race and ethnicity (White), and treatment (chemotherapy). Horizontal lines on dot plot represent 95% CIs with blue dots indicating statistical significance at *P* < .05. ICI indicates immune checkpoint inhibitors; LDL, low-density lipoprotein.

In multivariable Fine-Gray models adjusting for demographic and clinical factors, CHIP remained significantly associated with increased risk of heart failure (subdistribution hazard ratio [sHR], 1.26; 95% CI, 1.02-1.56; *P* = .03). Associations with ischemic CVD (sHR, 1.15; 95% CI, 0.95-1.38; *P* = .14) and arrhythmia (sHR, 1.02; 95% CI, 0.87-1.18; *P* = .85) were not statistically significant, although point estimates suggested a potential increased risk (Figure 2).

### Chemotherapy Intensity Analysis

**Figure 3.**
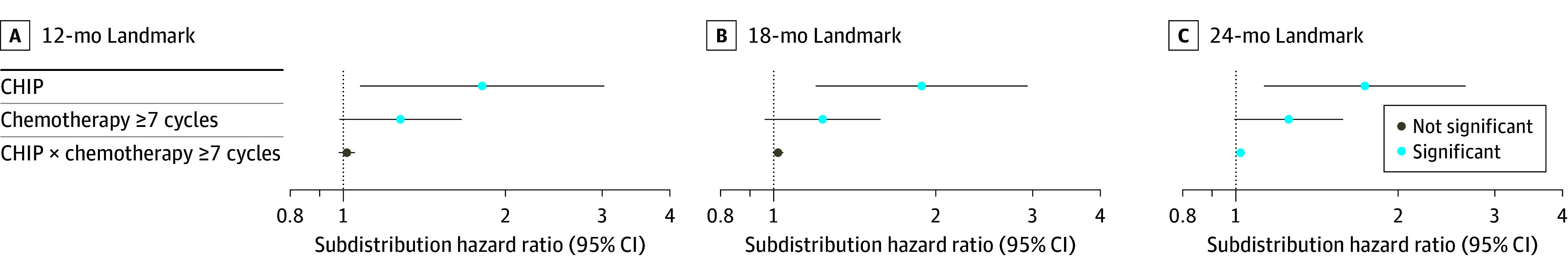
Landmark Analysis of Heart Failure Risk Associated With Clonal Hematopoiesis of Indeterminate Potential (CHIP) by Number of Chemotherapy Cycles After Matching Horizontal lines on dot plot represent 95% CIs with blue dots indicating marginal significance at *P* < .001.

Cumulative exposure to chemotherapy (see eTable 2 in Supplement 1 for a list of regimens) modified the association between CHIP and heart failure risk. In an exploratory 24-month landmark analysis, there was a statistically significant interaction between CHIP and intensive chemotherapy (≥7 cycles) and heart failure risk (sHR, 1.02; 95% CI, 1.00-1.04; *P* = .03). Both the main associations of CHIP (sHR, 1.73; 95% CI, 1.13-2.65; *P* = .01) and intensive chemotherapy (sHR, 1.25; 95% CI, 0.99-1.58; *P* = .057) were marginally significant (Figure 3).

## Discussion

To our knowledge, we performed the largest study to date testing the association between CHIP and CVD in patients with solid tumors treated with chemotherapy, radiotherapy, or immunotherapy. CHIP was associated with statistically significantly higher cardiovascular risk. Our exploratory landmark analysis at 24 months showed a statistically significant interaction between CHIP and the intensive chemotherapy, suggesting a potential amplifying effect that warrants further investigation in future studies. This aligns with findings from a prior study^19^ of 236 patients with solid tumors in a cardio-oncology clinic, which reported a 2-fold increased risk of cancer therapy–associated cardiomyopathy in those with CHIP. Notably, this risk exceeded the association observed in individuals with CHIP not exposed to cancer therapy, where prior population-based studies have reported hazard ratios for heart failure around 1.25.^20^ Consistent with this, in our analysis of individuals without cancer in BioVU, CHIP was associated with a more modest increased risk of heart failure (sHR, 1.17; 95% CI, 1.00-1.37; *P* = .04), highlighting a potential amplifying effect of chemotherapy in CHIP-associated CVD.

Unlike in individuals without cancer, *TET2* was not significantly associated with risk of incident heart failure; however, future work with larger sample sizes is required to interrogate genotype-specific findings.

The clinical implications of these findings are 2-fold. First, there may be value in testing patients for CHIP prior to cancer treatment initiation. CHIP status, along with established CVD risk factors, could be incorporated into a CVD risk stratification strategy tailored for cancer survivors. Second, patients with CHIP may benefit from more aggressive cardiovascular monitoring, earlier cardio-oncology consultation, or consideration of cardioprotective strategies.

### Limitations

This study had several limitations. First, we only analyzed data from a single biobank. We considered external validation in other large biobanks such as All of Us and the UK Biobank; however, the number of individuals with solid tumor with both CHIP and chemotherapy data in these biobanks were 155 and 36, respectively, limiting statistical power. Second, we used whole-genome sequencing, not deep targeted sequencing, to ascertain CHIP status and assumed that individuals with CHIP detected at sequencing also had CHIP when they initiated therapy. Third, we recognize that different treatment types vary in their cardiotoxic potential; thus, grouping treatments together was a limitation. However, given that only 6.9% of participants had CHIP, stratifying by individual treatment types would have yielded too few samples to obtain reliable estimates. Fourth, the limited number of participants with CHIP constrained analyses stratified by cancer type or heart failure subtype (HFpEF vs HFrEF). Fifth, we were unable to determine how cancer therapy impacted CHIP clonal dynamics because the cohort was sequenced only once. Understanding the interaction between clonal dynamics and cardiovascular risk in patients with cancer is an important area for future investigation. Future work should also compare CHIP-associated CVD risk between individuals with and without cancer to clarify whether cancer status or cancer therapies modify the cardiovascular effects of CHIP.

## Conclusions

In this cohort study, CHIP was associated with increased heart failure risk in patients receiving cardiotoxic cancer therapies. These findings support the potential clinical utility of CHIP testing for cardiovascular risk stratification for patients with solid tumors undergoing cancer treatment and suggest that incorporating CHIP status may improve cardio-oncology treatment of cancer survivors.

## Notes

Supplement 1. eTable 1. Variable Definitions for Diseases and Cancer Therapies eTable 2. Overview of Demographic and Clinical Profiles eTable 3. Multivariable Analysis of CVD after Propensity Score Matching eTable 4. Percentage of Missing Data by CHIP Status eFigure 1. Covariate Balance after 1:10 and 1:5 Propensity Score Matching eFigure 2. Association Between Time to Heart Failure and Five-Most Prevalent CHIP Genes

Supplement 2. Data Sharing Statement
