# Supplementary material for: Clonal Hematopoiesis and Cardiovascular Disease Risk After Cancer Therapy in Patients With Solid Tumors
Source: JAMA Oncol. 2026 Jan 8;12(3):251–6. doi: 10.1001/jamaoncol.2025.5785 (PMC12784258; doi:10.1001/jamaoncol.2025.5785)
Supplement: Supplement 1. — eTable 1. Variable Definitions for Diseases and Cancer Therapies eTable 2. Overview of Demographic and Clinical Profiles eTable 3. Multivariable Analysis of CVD after Propensity Score Matching eTable 4. Percentage of Missing Data by CHIP Status eFigure 1. Covariate Balance after 1:10 and 1:5 Propensity Score Matching eFigure 2. Association Between Time to Heart Failure and Five-Most Prevalent CHIP Genes [file jamaoncol-e255785-s001.pdf]

Supplemental Online Content

Shyr D, Pershad Y, Zhao K, et al. Clonal hematopoiesis and cardiovascular disease risk after cancer therapy in patients with solid tumors. *JAMA Oncol.* Published online January 8, 2026. doi:10.1001/jamaoncol.2025.5785

eTable 1. Variable Definitions for Diseases and Cancer Therapies
eTable 2. Overview of Demographic and Clinical Profiles
eTable 3. Multivariable Analysis of CVD after Propensity Score Matching
eTable 4. Percentage of Missing Data by CHIP Status
eFigure 1. Covariate Balance after 1:10 and 1:5 Propensity Score Matching
eFigure 2. Association Between Time to Heart Failure and Five-Most Prevalent CHIP Genes

This supplemental material has been provided by the authors to give readers additional information about their work.

© 2026 Shyr D, et al. *JAMA Oncology*.

eTable 1. Variable Definitions for Diseases and Cancer Therapies

**Diseases**	**ICD-09 and ICD-10 Codes**
**Cardiovascular Disease**	
Heart Failure	398.91, 402.01, 402.11, 402.91, 404.01, 404.03, 404.11, 404.13, 404.91, 404.92,<br>404.93, 428, 428.1, 428.2, 428.21, 428.22, 428.23, 428.3, 428.31, 428.32, 428.33, 428.4, 428.41, 428.42, 428.43, 428.9, I09.81, I11.0, I13.0, I13.2, I50, I50.1, I50.2, I50.20, I50.21, I50.22, I50.23, I50.3, I50.30, I50.31, I50.32, I50.33, I50.4, I50.40, I50.41, I50.42, I50.43, I50.8, I50.81, I50.810, I50.811, I50.812, I50.813, I50.814, I50.82, I50.83, I50.84, I50.89, I50.9
Ischemic cardiovascular disease	MI: I211, I210, I212, I213, I214, I219<br>Angina: I209<br>Other ischemic CVD: I249, I258, I259; Atherosclerosis: I250, I251, I252, I700, I709; Ischemic heart failure: I255; Ischemic stroke: I630, I631, I632, I633, I634, I635, I639, I672, I638, I693

© 2026 Shyr D, et al. *JAMA Oncology*.

Arrythmia	426, 426.1, 426.11, 426.12, 426.13, 426.2, 426.3, 426.4, 426.5, 426.51, 426.52,<br>426.53, 426.54, 426.6, 426.7, 426.8, 426.81, 426.82, 426.89, 426.9, 427, 427.1,<br>427.2, 427.3, 427.31, 427.32, 427.4, 427.41, 427.42, 427.6, 427.61, 427.69, 427.8, 427.81, 427.89, 427.9, 779.81, 779.82, 785, 785.1, I44, I44.0, I44.1, I44.2, I44.3, I44.30, I44.39, I44.4, I44.5, I44.6, I44.60, I44.69, I44.7, I45,<br>I45.0, I45.1, I45.10, I45.19, I45.2, I45.3, I45.4, I45.5, I45.6, I45.8, I45.81,<br>I45.89, I45.9, I47, I47.0, I47.1, I47.2,<br>I47.20, I47.21, I47.29, I47.9, I48,<br>I48.0, I48.1, I48.11, I48.19, I48.2,<br>I48.20, I48.21, I48.3, I48.4, I48.9,<br>I48.91, I48.92, I49, I49.0, I49.01,<br>I49.02, I49.1, I49.2, I49.3, I49.4, I49.40, I49.49, I49.5, I49.8, I49.9, P29.11,<br>P29.12, Q24.6, R00, R00.0, R00.1, R00.2,<br>R00.8, R00.9
**Solid Tumor**	
Breast	C500, C501, C502, C503, C504, C505, C506, C508, C509
Colorectal/Anorectal	C180, C181, C182, C183, C184, C185, C186, C187, C188, C189, C20, C210, C211, C212, C218, C170, C171, C173, C178, C179
Esophageal	C150, C151, C152, C153, C154, C155, C158, C159
Stomach	C160, C161, C162, C163, C164, C165, C166, C168, C169
Liver	C220, C221, C222, C223, C224, C227, C229, C230, C240, C241, C248, C249
Pancreas	C250, C251, C253, C254, C257, C258, C259
Prostate	C61

© 2026 Shyr D, et al. *JAMA Oncology*.

Men's Genitourinary System	C620, C621, C629, C630, C631, C632, C637, C638, C639, C600, C601, C602, C608, C609
Women's Genitourinary System	C530, C531, C538, C539, C510, C511, C512, C518, C519, C52, C56, C570, C571, C572, C573, C574, C577, C578, C579, C540, C541, C543, C548, C55
Renal	C64, C65
Bladder	C66, C670, C671, C672, C673, C674, C675, C676, C677, C678, C679, C680, C681, C688, C689
Lung	C340, C341, C342, C343, C349
Thyroid	C430
Melanoma	C64, C431, C432, C434, C435, C436, C437, C438, C439
**Myeloid Malignancies**	
Myelodysplastic Syndrome (MDS)	D460, D461, D462, D463, D464, D465, D466, D467, D469
Acute Myeloid Leukemia (AML)	C920, C923, C924, C925, C926, C930, C937, C939, C940, C942, C943, C944, C950
Myeloproliferative Neoplasm (MPN)	D45, D473, C946, D474
Chronic and Other Myelogenous Leukemia	C922, C931, C932, C941, C951, C952, D475, C957, C947, C937, C927, C959, C929
**Cancer Therapies**	

© 2026 Shyr D, et al. *JAMA Oncology*.

Chemotherapy	**RxNorm Codes:** 730548, 905078, 955632, 1301267, 1301597, 1304107, 1304919, 1305058, 1308290, 1309161, 1309188, 1310317, 1311078, 1311409, 1311443, 1311799, 1314865, 1314924, 1315942, 1315946, 1318011, 1319193, 1325363, 1326481, 1329241, 1333357, 1333379, 1336539, 1336825, 1337620, 1337651, 1338512, 1341149, 1343346, 1344354, 1344905, 1349025, 1350066, 1350504, 1351779, 1355509, 1355513, 1358436, 1359548, 1363387, 1367268, 1368823, 1377141, 1378382, 1378509, 1389036, 1389888, 1390051, 1391846, 1394023, 1394337, 1395557, 1397599, 1436650, 1437379, 1511250, 1750928, 19002912, 19008264, 19009165, 19011440, 19012543, 19012585, 19015523, 19024728, 19025348, 19031224, 19054821, 19054825, 19078097, 19078187, 19092845, 19136210, 19136750, 19137385, 35602757, 35604205, 35604657, 35605522, 35605804, 35606214, 40166461, 40167554, 40168385, 40222431, 40230712, 40238052, 40239056, 40241937, 40242675, 40244266, 40244464, 42709321, 42709322, 42873638, 42900401, 42903460, 43012292, 43013182, 43532299, 43532497, 43533090, 44507848, 44818466, 45775396, 45776670, 45776944, 45892075, 45892579, 46221433
Immune Checkpoint Inhibitors	**RxNorm Codes:** 45775965, 45892628, 42629079, 1593273, 1594034, 741851, 35200783, 40238188

© 2026 Shyr D, et al. *JAMA Oncology*.

Radiotherapy	**CPT Codes:** 0394T, 0395T, 77316, 77317, 77318, 77761, 77762, 77763, 77767, 77768, 77770, 77771, 77772, 77778, 77789, 77790, 77799, C9726, G0458, 0745T, 0746T, 0747T, 77371, 77372, 77373, 77432, 77435, G0339, G0340, 77301, 77338, 77385, 77386, G6015, G6016, 77423, 19294, 77424, 77425, 77469, 77520, 77522, 77523, 77525, S8030, 77600, 77605, 77610, 77615, 77620, 77427, 77431, 77470, 77499, 77261, 77262, 77263, 77280, 77285, 77290, 77293, 77401, 77402, 77407, 77412, 77417, A9609, C9794, C9795, G6003, G6004, G6005, G6006, G6007, G6008, G6009, G6010, G6011, G6012, G6013, G6014, 77014, 77387, G6001, G6002, G6017, 77295, 77300, 77306, 77307, 77321, 77331, 77332, 77333, 77334, 77336, 77370, 77399, 77750, 79005, 79101, 79403, A9513, A9543, A9606, A9607, A9590, A9699, C2616, S2095, 19296, 19297, 19298, 31643, 32553, 41019, 49411, 49412, 55875, 55876, 55920, 57155, 57156, 58346, 76873, 76965, 61796, 61797, 61798, 61799, 61800

eTable 2. Overview of demographic and clinical profiles. Continuous and categorical variables were summarized as median (Q1, Q3) and n (%), respectively.

**Characteristic**	**No CHIP**<br>N = 7,455	**CHIP**<br>N = 549
Age (years)	61.3 (51.6, 69.3)	69.8 (62.3, 75.7)
Gender		
Female	4,134 (55%)	251 (46%)
Male	3,321 (45%)	298 (54%)

© 2026 Shyr D, et al. *JAMA Oncology*.

Race		
Asian	82 (1.1%)	4 (0.7%)
Black	842 (12%)	53 (9.8%)
Indian/Alaskan	9 (0.1%)	0 (0%)
White	6,358 (87%)	484 (89%)
Ethnicity		
Hispanic	166 (3.0%)	11 (2.6%)
Not Hispanic	5,458 (97%)	420 (97%)
Ever Smoked		
No	4,707 (63%)	324 (59%)
Yes	2,748 (37%)	225 (41%)
Hypertension		
No	2,244 (31%)	120 (22%)
Yes	5,086 (69%)	418 (78%)
LDL	102.7 (95.0, 104.5)	102.7 (88.0, 102.7)
Primary Solid Tumor		
Breast	2,349 (32%)	118 (21%)
Gastrointestinal	2,106 (28%)	154 (28%)
Genitourinary	2,248 (30%)	217 (40%)
Lung	141 (1.9%)	17 (3.1%)
Melanoma	607 (8.1%)	42 (7.7%)
Thyroid	4 (<0.1%)	1 (0.2%)
Chemotherapy Type		
Alkylating Agent/ Mitomycin	209 (3.8%)	14 (3.5%)

© 2026 Shyr D, et al. *JAMA Oncology*.

Anthracycline	361 (6.6%)	22 (5.5%)
Nitrogen Mustard	400 (7.3%)	27 (6.7%)
Other	1,194 (22%)	90 (22%)
Platinum Compound	1,085 (20%)	73 (18%)
Pyrimidine Analog	962 (17%)	92 (23%)
Taxane	1,065 (19%)	69 (17%)
TKI	226 (4.1%)	14 (3.5%)
Chemotherapy No. Cycles	5 (2, 12)	5 (2, 10)
Chemotherapy		
No	1,953 (26%)	148 (27%)
Yes	5,502 (74%)	401 (73%)
Radiotherapy		
No	3,784 (51%)	288 (52%)
Yes	3,671 (49%)	261 (48%)
Immune Checkpoint Inhibitors		
No	6,936 (93%)	517 (94%)
Yes	519 (7.0%)	32 (5.8%)
Ischemic CVD		
No	5,712 (83%)	359 (75%)
Yes	1,203 (17%)	118 (25%)
Heart Failure		
No	6,056 (87%)	412 (82%)
Yes	878 (13%)	92 (18%)
Arrhythmia		

© 2026 Shyr D, et al. *JAMA Oncology*.

No	3,302 (59%)	220 (57%)
Yes	2,315 (41%)	165 (43%)

eTable 3. Multivariable Analysis of CVD after Propensity Score Matching. Reference levels of categorical covariates include: *Sex (Female), Race/Ethnicity (White), Treatment (Trt: Chemotherapy)*; sHR = subdistribution hazard ratio.

Variable	Heart Failure, sHR (95% CI)	Ischemic Stroke, sHR (95% CI)	Arrhythmia, sHR (95% CI)
CHIP	1.201 (0.970–1.487)	1.055 (0.876–1.272)	1.002 (0.859–1.169)
Age	1.022 (1.016–1.028)	1.035 (1.030–1.041)	1.007 (1.003–1.010)
Sex: Male	1.249 (1.096–1.425)	1.482 (1.324–1.659)	1.181 (1.087–1.283)
Race/Ethnicity: Black	1.391 (1.155–1.675)	1.301 (1.101–1.537)	1.200 (1.061–1.358)
Race/Ethnicity: Hispanic	0.569 (0.298–1.085)	0.890 (0.553–1.433)	0.999 (0.739–1.352)
Race/Ethnicity: Other	0.462 (0.171–1.250)	0.766 (0.378–1.552)	1.106 (0.739–1.655)
Trt: Radiation	0.676 (0.562–0.813)	0.823 (0.708–0.957)	0.786 (0.700–0.883)
Trt: ICI	0.385 (0.182–0.816)	0.504 (0.280–0.908)	0.628 (0.427–0.924)
Trt: Chemo + Rad	1.050 (0.905–1.218)	1.090 (0.960–1.237)	1.323 (1.207–1.451)
Trt: Chemo + ICI	1.794 (1.206–2.669)	1.027 (0.642–1.642)	1.718 (1.288–2.290)

© 2026 Shyr D, et al. *JAMA Oncology*.

Trt: Rad + ICI	0.852 (0.431–1.685)	0.764 (0.402–1.454)	1.825 (1.268–2.626)
Trt: Chemo + Rad + ICI	1.538 (1.078–2.195)	1.261 (0.887–1.792)	1.890 (1.514–2.359)
Hypertension	2.998 (2.417–3.719)	2.778 (2.308–3.345)	1.725 (1.563–1.905)
LDL Cholesterol	1.003 (0.993–1.006)	1.003 (0.995–1.006)	1.006 (0.999–1.010)

eTable 4. Percentage of Missing Data by CHIP Status

Variable	No CHIP	CHIP	Overall
LDL	1.7%	1.9%	1.7%
Hypertension	1.7%	1.9%	1.7%

eFigure 1. Covariate Balance after 1:10 and 1:5 Propensity Score Matching. Blue circles represent matched while grey triangles unmatched

© 2026 Shyr D, et al. *JAMA Oncology*.

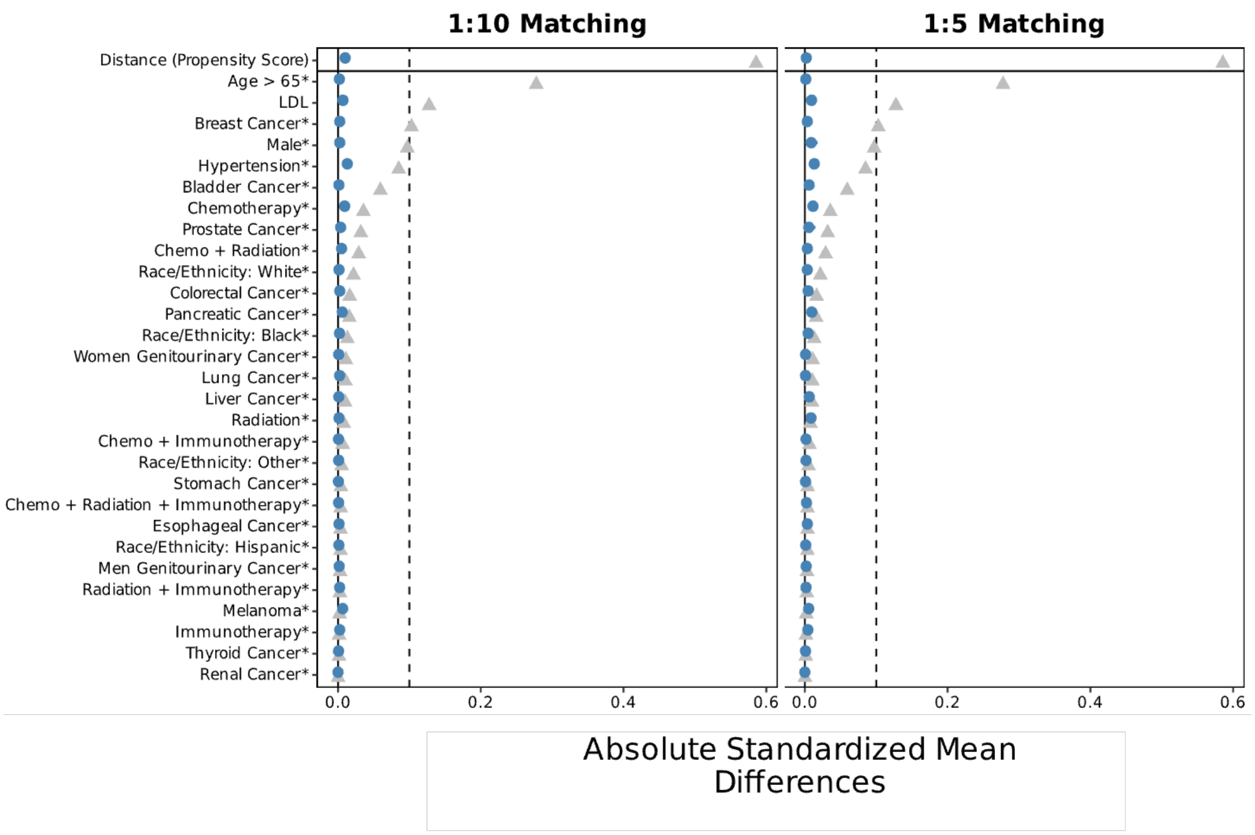

eFigure 2 . Association Between Time to Heart Failure and Five-Most Prevalent CHIP Genes. Horizontal lines on forest plot represent 95% confidence interval with blue indicating statistical significance at P < 0.05.

© 2026 Shyr D, et al. *JAMA Oncology*.

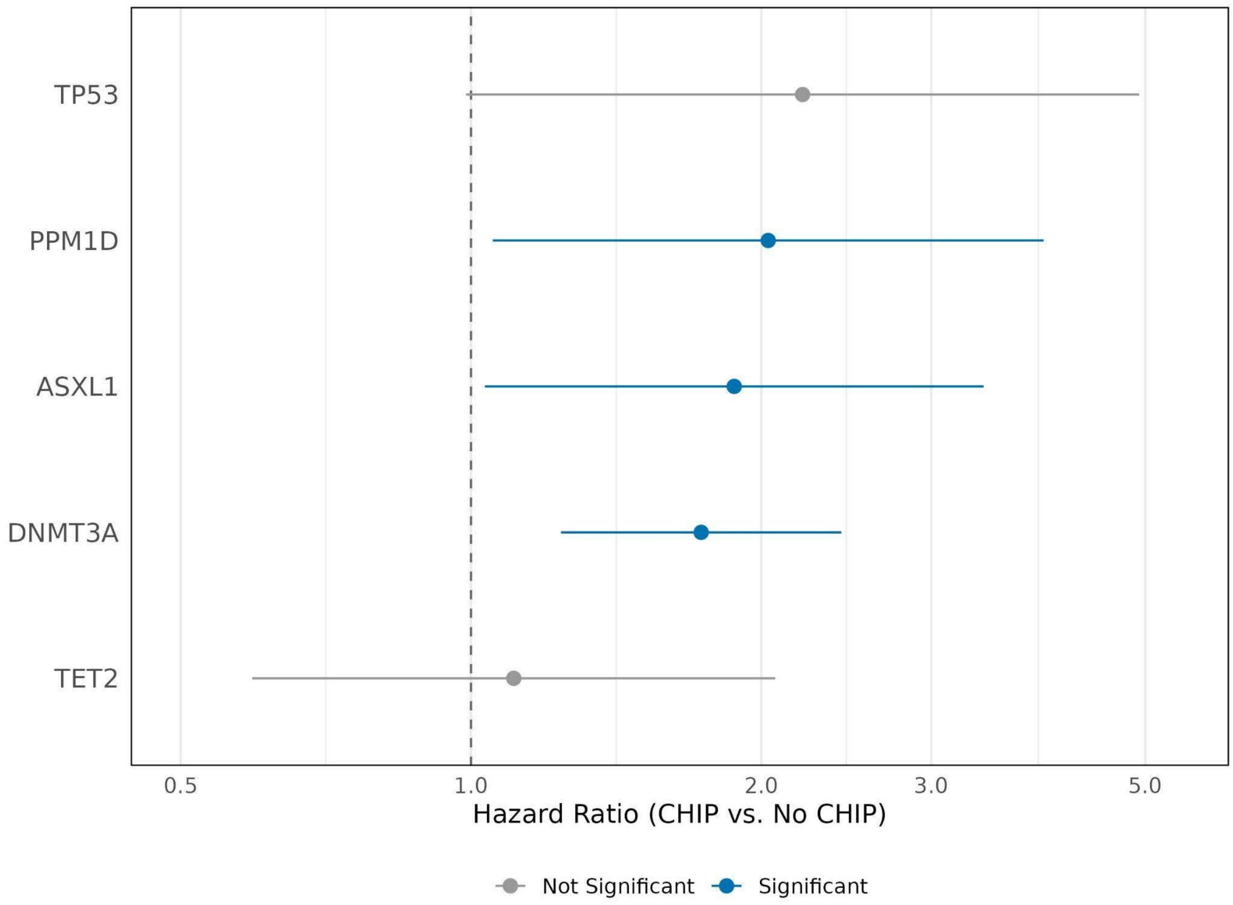

© 2026 Shyr D, et al. *JAMA Oncology*.
