# Supplementary material for: Clonal Hematopoiesis and Cardiovascular Disease Risk After Cancer Therapy in Patients With Solid Tumors
Source: JAMA Oncol. 2026 Jan 8;12(3):251–6. doi: 10.1001/jamaoncol.2025.5785 (PMC12784258; doi:10.1001/jamaoncol.2025.5785)
Supplement: Supplement 2. — Data Sharing Statement [file jamaoncol-e255785-s002.pdf]

Data Sharing Statement

Shyr. Clonal Hematopoiesis and Cardiovascular Disease Risk after Cancer Therapy in Patients With Solid Tumors. *JAMA Oncol*. Published January 08, 2026.
doi:10.1001/jamaoncol.2025.5785

Data
Data available: Yes
Data types: Deidentified participant data, Data dictionary
How to access data: biovu@vumc.org
When available: With publication

Supporting Documents
Document types: None

Additional Information
Who can access the data: Researchers whose proposed use of the data has been approved
Types of analyses: For purposes of replicating the original analyses
Mechanisms of data availability: with a signed data access agreement
